# Spatial Competition: Roughening of an Experimental Interface

**DOI:** 10.1038/srep29908

**Published:** 2016-07-28

**Authors:** Andrew J. Allstadt, Jonathan A. Newman, Jonathan A. Walter, G. Korniss, Thomas Caraco

**Affiliations:** 1 Department of Forest and Wildlife Ecology, University of Wisconsin-Madison, Madison, Wisconsin 53706 USA; 2 College of Biological Science, University of Guelph, Guelph, Ontario N1G 2W1 Canada; 3 Department of Ecology and Evolutionary Biology and Kansas Biological Survey, University of Kansas, Lawrence, KS 66047 USA; 4 Department of Physics, Applied Physics and Astronomy, Rensselaer Polytechnic University, Troy, NY 12308 USA; 5 Department of Biological Sciences, University at Albany, Albany, New York 12222 USA

## Abstract

Limited dispersal distance generates spatial aggregation. Intraspecific interactions are then concentrated within clusters, and between-species interactions occur near cluster boundaries. Spread of a locally dispersing invader can become motion of an interface between the invading and resident species, and spatial competition will produce variation in the extent of invasive advance along the interface. Kinetic roughening theory offers a framework for quantifying the development of these fluctuations, which may structure the interface as a self-affine fractal, and so induce a series of temporal and spatial scaling relationships. For most clonal plants, advance should become spatially correlated along the interface, and width of the interface (where invader and resident compete directly) should increase as a power function of time. Once roughening equilibrates, interface width and the relative location of the most advanced invader should each scale with interface length. We tested these predictions by letting white clover (*Trifolium repens*) invade ryegrass (*Lolium perenne*). The spatial correlation of clover growth developed as anticipated by kinetic roughening theory, and both interface width and the most advanced invader’s lead scaled with front length. However, the scaling exponents differed from those predicted by recent simulation studies, likely due to clover’s growth morphology.

In many plant communities, limited dispersal aggregates conspecific individuals^1^. In particular, most invasive plants are clonal and propagate vegetatively^2^, so that invaders initially cluster among residents^3^. Aggregation of conspecifics has consequences for population interactions. Individual plants usually compete at the nearest-neighbor scale^4^,^5^. When different species each aggregate spatially and interact locally, intraspecific competition will predominate within clusters, while interspecific competition will localize at the interface between clusters^6^,^7^,^8^. This interaction geometry implies that the advance *versus* extinction of an invasive species may depend on development and subsequent movement of a between-species interface^9^,^10^.

An invading species’ local density declines from positive equilibrium to rarity across the width of an ecological interface^11^. As a competitively superior invader excludes the resident species within the interface width, the front is pushed forward. Dispersal limitation promotes spatially correlated invasive advance along the interface. These correlations, generated through lateral growth, invite application of the theory of kinetic roughening, a framework for identifying quantitative characteristics shared by different interface-growth processes^12^. Previous applications of the theory span materials science^13^, temporal pattern in parallel-computing^14^,^15^, and ecological invasion^11^,^16^.

Kinetic roughening theory predicts power-law scaling relationships governing both the development and the equilibrium statistical structure of an invader-resident interface. Our analyses emphasize scaling of both the interface width and the relative position of the “front-runner,” the most advanced invader, a metric used at both local and regional scales^17^,^18^,^19^. Interestingly, the exponents of scaling relationships predicted by kinetic roughening sometimes identify an interface as a member of a particular universality class. That is, quite distinct local processes may exhibit the same dependence of interface roughening on time, and the equilibrium width may exhibit the same dependence on interface length; universality implies powerful generality^13^. Previously, we modeled the front produced when a dispersal limited, but competitively superior, invader advances across a habitat occupied by a resident species^11^,^20^. That model’s kinetic roughening belongs to the KPZ universality class, for Kardar-Parisi-Zhang^12^.

We begin by analyzing spatial competition as a problem for kinetic roughening theory, and then report a field experiment testing the predictions. We let Dutch white clover (*Trifolium repens*) advance into plots of perennial ryegrass (*Lolium perenne*). We monitored the development of spatial correlations along the fronts, and estimated a series of power-law scaling relationships from roughened fronts of different lengths. The exponents implied by the observed scaling allowed us, in addition, to ask if the experimental interface belonged to the KPZ universality class^12^,^13^.

## Local Dispersal and Interface Roughening

A dispersal-limited invader’s population typically begins as one or more clusters of individuals. Although the invader may have a competitive advantage, some small clusters will disappear due to demographic stochasticity. But clusters exceeding a critical size will continue to grow and displace the resident^21^. After a single cluster attains sufficient size, or after large clusters coalesce, we can treat the perimeter as a 1-dimensional front that has roughened during advance^22^. Some invasions move perpendicularly to a road or shore; they can be treated as initially linear^16^. The dynamics of an ecological interface distinguishes it from an ecotone, since the latter implies a change in species composition due to abiotic factors that vary slowly relative to the timescale of population growth^23^,^24^.

Our analysis treats the invader as competitively superior to the resident. We assume that the ecological interface, once roughening equilibrates, has an anisotropic fractal geometry^25^. This assumption has quantitative ecological implications; both interface width and the front-runner’s lead should increase as a power function of the length of the advancing front^20^. When we analyze the front-runner’s location, correlated fluctuations along the interface are important, since traditional extreme-value statistics^26^,^27^, developed for *independent* random variables, do not apply^28^.

**Figure 1:**
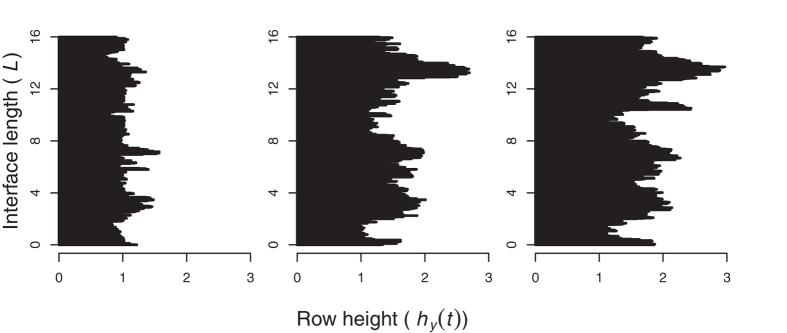
White clover (*T. repens*, black area) advancing into perennial ryegrass (*L. perenne*), from photographs taken during experiment. Interface length *L* ≡ *L*_*y*_ = 16 *m*. June (left), August (center) and October (right) 2010 shown. The interface advances, left to right, and roughens; neighboring heights suggest spatial correlation.

Figure 1 shows an interface from our field experiment. The extent of invasive growth along the interface clearly becomes more variable, i.e. roughens, with time. The plots also suggest correlated advance at nearby locations.

### Interface Roughening: Development and Saturation

After defining interface attributes for spatial competition between species, we describe development of a roughened interface. Then we address scaling relationships at equilibrium (after roughening “saturates”). Table 1 lists symbols we use.

Discrete (“individual-based”) models capture effects of nonlinearity and stochasticity inherent to a dispersal-limited invader’s dynamics at an ecological interface^29^,^30^. Therefore, we characterize invasion as a lattice process; our description applies across a variety of individual-based models for growth at an interface^20^,^31^. An *L*_*x*_ × *L*_*y*_ rectangular lattice represents a habitat occupied by resident and invader species. Each lattice site is either occupied by the invader, occupied by the resident, or empty. Mortality of either species opens occupied sites. An empty site becomes occupied through propagation from a nearest-neighboring occupied site. The same dispersal limitation applies to both invader and resident, precluding a competition-colonization trade-off that permits species‘ coexistence^32^,^33^. Then the invader’s competitive superiority drives interface motion. If invader-resident competition is preemptive only, the invader has the lower mortality/propagation ratio^34^. If competition combines site preemption and interference competition, exclusion of the resident can require that the invader’s propagation rate be great enough to overcome the combined effect of resident propagation and increased mortality due to resident interference^35^.

Suppose that the invader initially occupies a few vertical columns at the left edge of the lattice; all other sites are occupied by the resident or open. Invasive advance occurs in the *x*-direction. Importantly, the dispersal constraint permits both forward and lateral growth. The former pushes the front, and the latter, at advanced heights, generates spatial correlation along the front^12^,^13^.

We let *L* ≡ *L*_*y*_, interface length (hence, front length). At time *t, h*_*y*_(*t*) is the location of the most advanced (right-most) invader in row *y; y* = 1, 2, …, *L*. The front’s average location is the mean height among rows, 
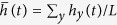
. We take longitudinal system size *L*_*x*_ as sufficiently large that it does not affect population processes.

We define the width of the interface *via* roughness:


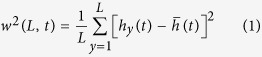


**Figure 2:**
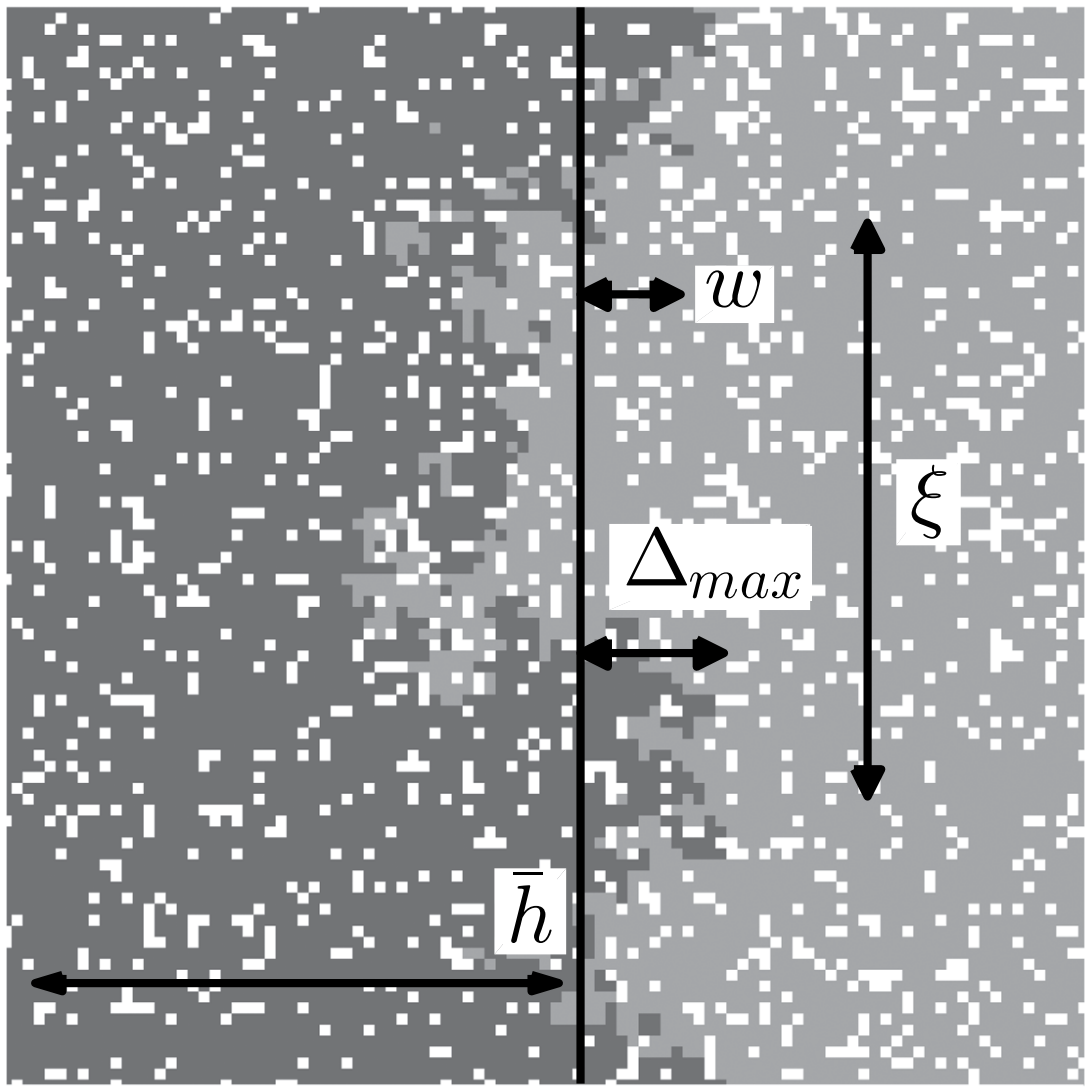
Schematic plot of the width (*w*) and the extreme advance (Δ_*max*_) relative to the mean front position 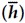 in a rough front. For illustration, correlation length *ξ* is also indicated. Dark: invader, medium: resident, and white: open.

Roughness *w*^2^(*L, t*) itself varies stochastically, and we represent its expectation (averaged over realizations of intrinsic noise) at time *t* by 〈*w*^2^(*L, t*)〉. We take 
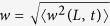
 as width of the front, the typical extent of the interface parallel to the direction of advance. Figure 2 diagrams a between-species interface and shows the width about the invader’s average incursion 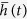.

The development of spatially correlated interface heights underlies the power-law scaling we address. These scaling relationships do not, in general, depend on details of the local growth dynamics^25^. That is, despite variation in demographic details among different invader-resident interactions, the same qualitative scaling patterns should emerge^16^,^20^. Spatially correlated heights imply that the interface should equilibrate as a statistically self-affine fractal; this structure produces ecologically interesting scaling laws. A roughened, self-affine interface has a width *w*(*L*), where *L* is front length. Suppose that we increase length according to *L*→*kL*. Then interface width must be re-scaled according to *w*→*k*^*α*^*w* to preserve statistical invariance (“look the same” at different scales). Length and width must be increased by different factors, and the transformation has a power-law form. Numerical calibration of the scaling laws can, of course, differ across species and environments.

### Interface Development

As the invader begins to advance, the interface starts to roughen, and invader heights *h*_*y*_(*t*) become dependent random variables. A single correlation length *ξ*(*t*) develops along the interface (Fig. 2). Correlation length initially increases with time according to the power-law scaling *ξ*(*t*) ~ *t*^1/*z*^; *z* is called the dynamic exponent^13^,^36^. Once *ξ*(*t*) spans the length *L* of the interface, “crossover” occurs. The interface continues to advance, but roughening has reached statistical equilibrium (roughening “saturates” at crossover)^13^. The duration of interface development, termed crossover time *t*_×_, increases with interface length; the scaling is *t*_×_ ~ *L*^*z*^. The development of interface width offers a more useful ecological prediction. Prior to saturation, interface width *w* expands with time according to *w* ~ *t*^*β*^. *β (β* > 0) is called the growth exponent.

The height-height correlation function (Pearson correlation) is:


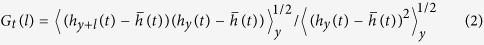


where the averages are taken across rows *y. G*_*t*_(*l*) indicates correlation length *ξ*(*t*) along the interface; height-height correlation should decline as distance *l* between rows increases.

We use two statistics to monitor roughening associated with increasing correlation length along a developing interface. Each combines results from windows of length *l* < *ξ*(*t*). The local width *w*_*t*_(*l*) averages interface widths from an ensemble of portions of the interface, each of length *l*. The local width is given by:


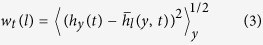


where 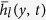 is the mean height in the local window of length *l*.

Second, the height-difference correlation function, at time *t* > 0, is given by:


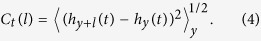


Both *w*_*t*_(*l*) and *C*_*t*_(*l*) are averaged across all rows *y*. For *l* < *ξ*(*t*), both *w*_*t*_(*l*) and *C*_*t*_(*l*) exhibit power-law scaling over distances along the interface: *w*_*t*_(*l*), *C*_*t*_(*l*) ~ *l*^*α*^. *α (α* > 0) is the roughness exponent, and characterizes the fractal nature of the interface^13^,^37^. As the interface roughens with time, the correlation distance *ξ* increases. Consequently, the linear dependence of ln *w*_*t*_(*l*) and ln *C*_*t*_(*l*) on ln *l*, with slope *α*, should extend to greater lengths *l* along the interface, until saturation.

### The Saturated Interface

After crossover (*t* > *t*_×_), steady-state properties of the interface depend on its length *L*^13^,^38^. Interface width *w* scales with interface length according to 〈*w*^2^(*L*, ∞)〉 ~ *L*^2*α*^; interface width increases as a power function of its length, according to the roughness exponent *α*.

Note that we do not predict roughness *per se*, but ask how interface roughness changes as we increase interface length. Power-law scaling for 〈*w*^2^(*L*, ∞)〉 permits us to ask novel questions about invasive spatial growth. Cannas *et al*.^16^ hypothesize that life-history variation among invading tree species might influence the roughness exponent. Our analysis emphasizes how the expected location of the invader’s extreme advance depends on the scaling of roughness with interface length.

### Scaling and the Front-Runner

Consider the maximal invasive advance, the front-runner’s position. At time *t* we locate the front-runner at *h*_*max*_(*t*) = max_*y*_{*h*_*y*_(*t*)}. Given mean interface height 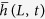, the invader’s maximal relative advance at time *t* is 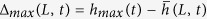. We assume that roughening equilibrates before considering the scaling of the expected lead 〈Δ_*max*_〉_*L*_; note dependence on interface length *L*.

The probability density of the front-runner’s excess Δ_*max*_(*L, t*) has been obtained analytically^28^,^36^. For broad classes of dispersal-limited stochastic growth models, the scaled variable Δ_*max*_/〈Δ_*max*_〉 has an Airy probability density, and the steady-state average excess of the front-runner over the mean height scales with interface length exactly as does the width. That is, 〈Δ_*max*_〉_*L*_ ~ *L*^*α*^. Furthermore, we can infer the size of the extremes for an interface of linear size *L* with estimates obtained in limited observation windows with size *l*. We have: 〈Δ_*max*_(*L*)〉 ≈ 〈Δ_*max*_(*l*)〉*k*^*α*^, where *k* = (*L*/*l*), by the properties of a self-affine interface. Table 2 collects the various scaling relationships we study.

### Scaling Exponents and KPZ Universality

In general, the scaling exponents of a roughened interface are dependent: *α* = *βz*^12^. Dependence arises from the fractal structure of the interface; that is, we assume that the interface equilibrates as a stochastically self-affine fractal^13^.

For roughened fronts in the KPZ universality class, the scaling-law exponents take particular values: *α* = 1/2, *β* = 1/3, and *z* = *α*/*β* = 3/2^14^. The continuous approximation underlying the KPZ-roughening includes terms for both frontal growth and lateral growth, and a term for additive, uncorrelated Gaussian noise^13^. A number of different interface-growth models belong to the KPZ universality class, and below we compare our results to the KPZ exponent values.

## Experimental Results

### Clover advance

**Figure 3:**
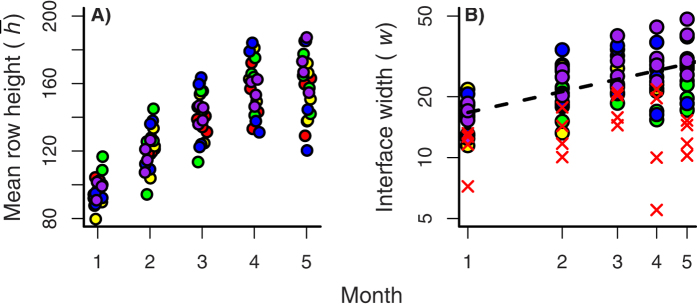
Experimental interface development. (**A**) Mean plot heights (*cm*) by month. Red, yellow, green, blue, and purple indicate, respectively, *L* = 1, 2, 4, 8, 16 *m*. “Noise” added to abscissa for visibility. (**B**) Each plot’s interface width by month; note the log-log scales. Dashed line indicates scaling during development. Estimated growth exponent *β* = 0.312. 1 *m* plots marked as X, signifying earlier saturation; see text.

During the 2010 growing season, clover advanced rapidly into the ryegrass; several longer fronts approached the far end of the plot by October. Figure 3A shows each plot’s mean height 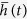 against time. Figure 3B shows the interface width *w* as a function of time for the same samples (to be discussed in detail in the Front Roughening subsection).

We estimated interface velocity as the difference in monthly mean clover height. Combining all plots, clover advanced fastest during the first month of growth. September velocities (after roughening saturated) were independent of interface length *L*. After September, longer fronts continued to advance, but some shorter fronts receded as the growing season ended. During the period of interface roughening, overall mean clover height advanced at 20.7 *cm*/*mo* (±0.33 *cm*/*mo*, SE). This exceeds mean stolen-elongation rates cited by Cain *et al*.^39^, but is within the range of clover “dispersal distances”^40^.

Overall mean clover height increased for five consecutive months. However, several clover fronts began to experience winter die-back in October. Therefore, our analysis treated data from June through August as the interface-development period, and treated data from September (month 4) as stationary. This is an approximation, since correlation lengths for larger values of *L* continued to increase during October.

### Spatial Correlation

**Figure 4:**
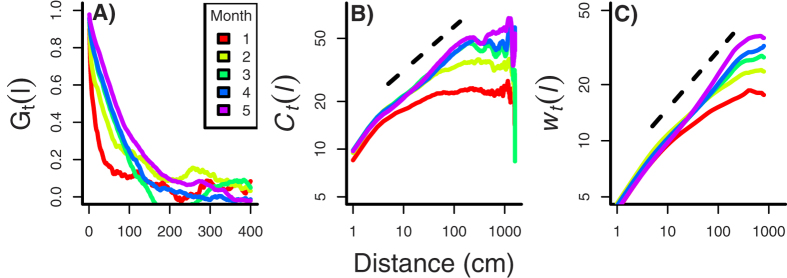
Field experiment: interface development. (**A**) Spatial correlogram: correlation of row heights, *G*_*t*_(*l*) [Eq. (2)]. The strength and lag distance at which correlations remained significant increased through time, indicating an increase in the correlation length, *ξ*(*t*), along the interface. Key indicates month 1 through 5 for each plot. (**B**) Height-difference correlation function, *C*_*t*_(*l*) [Eq. (4)], for months 1 through 5. Distance over which power-law scaling holds increases with time, that is, increases as correlation distance increases. The dashed line indicates the scaling relationship for month 4, based on the estimated growth exponent *α* = 0.277. (**C**) Local interface width *w*_*t*_(*l*) across months. The dashed line again shows the estimated scaling relationship for month 4 of *α* = 0.311. Note the log-log scales in (**B**,**C**).

Spatial correlations between row heights *h*_*y*_(*t*) both increased in strength and extended to greater distances along the interface as clover advanced. The initial development of correlation length should not depend on *L*, so we pooled observations from all plots. Each month we estimated correlation *G*_*t*_(*l*) between row heights *h*_*y*_(*t*) as a function of distance *l*. Figure 4A shows the correlogram; spatial correlation increased every month across most distances less than 200 *cm*.

The height-difference correlation *C*_*t*_(*l*) corroborated the previous result; see Fig. 4B. Each month *C*_*t*_(*l*) scaled as a power law for an increasing distance along the interface. Using the result for month 4, our model selection procedure strongly supported a power-law relationship with multiplicative error (Table 3). Power-law behavior of the height-difference correlation allows an estimate of the roughness exponent, since *C*_*t*_ ~ *l*^*α*^, for *l* < *ξ*(*t*). Regression analysis of the *C*_*t*_(*l*) results yielded *α* = 0.277; see Fig. 4B.

### Front Roughening

As the interface roughens, its width should increase with time according to 〈*w*^2^(*L, t*)〉^1/2^ ~ *t*^*β*^. Figure 3B shows each plot’s interface width against time. We tested the predicted scaling after excluding data for *L* = 1 *m*, since roughening in those (smallest) plots equilibrated earlier than observed for larger *L*. Model selection found support for the power-law model with multiplicative error (Table 3). Using this model, we estimated the growth exponent *β* as 0.312 (±0.073, SE); see Fig. 3B. Inclusion of plots where *L* = 1 *m* had little effect; the resulting estimate is *β* = 0.343 (±0.059, SE).

Figure 4C shows how local interface widths *w*_*t*_(*l*) increased as the interface developed. Each month *w*_*t*_(*l*) scaled as the same power law for a greater distance along the interface, as anticipated from the increase in correlation length *ξ*(*t*).

### Stationary Roughness and the Front-Runner

Assuming that roughening equilibrated in month 4, we tested the predicted scaling of interface width against alternatives in two ways. The first uses the local roughening analysis, and the second asks how mean interface width increases with *L*.

After saturation, local width *w*(*l*), where (*l* ≤ *L*), should scale as *w*(*l*) ~ *l*^*α*^. We combined month-4 data from different plots to characterize local roughening; see Fig. 4C. Our AIC-criterion (see Methods) strongly supported the power-law formulation with multiplicative error (Table 2). The associated estimate of the roughness exponent was *α* = 0.311.

**Figure 5:**
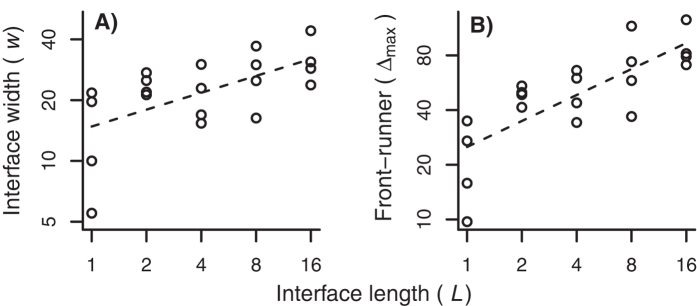
Saturated roughening and the front-runner. (**A**) Interface widths (*cm*)for different front lengths *L*, data from September (month 4). Dashed line indicates power-law scaling. (**B**) Front-runner’s exceedance (Δ_*max*_), in *cm*, for different interface lengths *L*, data from month 4. Dashed line indicates power-law scaling. Note the log-log scales in both panels.

The mean roughening analysis treated each plot’s width *w*(*L*) separately. Using September estimates (Fig. 5A), the model selection procedure again provided substantial support for a power-law relationship with multiplicative error (Table 3). The power-law model for mean interface width as a function of interface length gave an estimate *α* as 0.278 ± 0.181 (SE).

Once roughening has equilibrated, the average lead of the front-runner, beyond the mean height, should scale with length as 〈Δ_*max*_〉_*L*_ ~ *L*^*α*^. Our model selection procedure once again found support for power-law scaling with multiplicative error (Table 3). Using the preferred model, the front-runner scaling estimated the roughening exponent as *α* = 0.475 ± 0.091 (SE); *R*^2^ = 0.6; see Fig. 5B.

Table 2 lists our estimated scaling exponents. The length-based estimates of the roughness exponent *α* are consistent; scaling of the front-runner suggests greater roughness; we discuss this difference below. But every statistical analysis involving either the growth or the roughness exponent supported a power-law formulation over statistical alternatives, as follows from the assumed fractal structure of the interface.

## Ecological Implications

For dispersal-limited plants, ecological interactions driving invasive advance will often occur within the width of a between-species interface. The framework of kinetic roughening quantitatively organizes scaling effects produced by spatially correlated invasive growth. In turn, estimates of interface width and roughness help reveal the local structure of the within and between species interactions underlying spatial invasion.

As interface width, *w*_*t*_, increases during development, the area within which invader and resident individuals compete for space, *L* × *w*_*t*_, increases. Averaging invader density across rows *y* produces an interface profile, which summarizes the pattern of interactions within the width *w*_*t*_. Profiles for our experimental data show the fraction of *L* rows occupied by clover as a function of distance from the mean height at time *t*.

Figure S1 in the Supplementary Information shows interface profiles from one experimental plot (16 *m*, same as Fig. 1) for five months. Every month clover and ryegrass occurred with nearly equal frequency at the mean height. The first month’s (June) profile drops sharply; the competitors mix very little as the interface begins to develop. The remaining profiles show how interface width increases, which enlarges the area of interspecific mixing.

We approximated observed interface profiles with the complementary error function (see Supporting Information), since mean invader density has a roughly Gaussian decline across the interface^41^. If *ρ*_*t*_(*h*) represents clover density at height *h* and time *t*. Then:


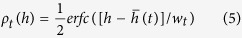


where *erfc* is the complementary error function, and *w*_*t*_ is interface width estimated at time *t. ρ*_*t*_(*h*) “flattens” as *w*_*t*_ increases. Increased interface width can decrease the frequency of the invader’s self-regulating interactions within the enlarging interface, and can increase the frequency of competitive interaction with the resident species. The Supplementary Information shows how these frequencies change as the width expands.

## Discussion

Given within-species spatial clustering, interspecific interactions will often occur at the interface where clusters contact. This depiction of plant spatial competition, common to numerous models, invites application of kinetic-roughening theory as a way to link pattern and process in dispersal-limited organisms. The framework requires only a few parameters to predict a series of scaling relationships applicable across a diverse local-growth processes. The methods of kinetic roughening should apply across physical scales if the growth processes are sufficiently similar. Perhaps, expansion or contraction of a species’ geographic range might be characterized as interface movement between habitats, driven by gain and loss of local demes. The obvious complication across greater distances is environmental heterogeneity^23^. Spatial heterogeneity in demographic rates, varying at a scale much greater than local dispersal distance, challenges application of kinetic roughening in analyzing ecological invasion. But generalizations of the scaling principles we invoked may prove useful for spatially heterogeneous invasion processes^13^.

In our field experiment, length-based estimates of the roughness exponent *α* were close to 0.3, and the scaling of the front-runner’s lead yielded an estimated *α* close to 0.5. Lattice models for clonal growth usually assume that an individual (ramet) propagates forward, backward and laterally; any unoccupied, nearest-neighboring site can be colonized at the same rate. But Cain *et al*.^39^ carefully mapped the architecture of clonal growth in a white clover population. Node-to-node branching angles of apical meristems centered on 0° (straight ahead), but some large angles were observed. Lateral meristems branched off with a bimodal angular distribution, concentrated at ±60–70°. Clover, then, exhibits forward and lateral growth, but with a bias toward forward propagation. The resulting morphology could have induced the difference between the scaling of the front-runner’s lead and the length-scaling of interface roughening.

Estimates of dynamic-scaling exponents abound for advancing fronts in physical systems^13^. In biology the framework has been invoked to model advancing fronts from bacterial colonies^42^,^43^ to forest expansion^16^, but seldom have the scaling exponents been measured empirically. A microscopic analysis of bacterial growth yielded two roughness exponents^44^. One experimental treatment promoted longer-range correlations along the front; estimated roughness was *α* = 0.78 ± 0.02 agreeing with an earlier, independent result^42^. The second estimate, involving a mutant bacterial strain and a different substrate, yielded a lesser value: *α* = 0.5 ± 0.01^44^. A dynamic-scaling study of callus growth, a proliferation of plant tissues in response to surface injury, produced an estimated roughness exponent of *α* = 0.86 ± 0.04^45^. Point estimates of the roughness exponent of our clover fronts, growing under interspecific competition for space, tended to be lower. However, standard errors of our estimates are relatively large. As a consequence, in 4 of 5 cases (see Table 2) we cannot reject the hypothesis of no difference between our estimated exponents and the KPZ values. In general, variation among both abiotic environments and local biotic interactions regulating growth may diversify interface geometries in biological systems^42^, and present a challenge to the application of organizing principles.

## Methods

### Spatial Competitors

We studied dispersal-limited competition between Dutch white clover (*T. repens*) and perennial ryegrass (*L. perenne*). Both species reproduce mainly through local, clonal growth^40^,^46^. *T. repens* propagates vegetatively through stoloniferous stems^47^, while *L. perenne* produces tillers^48^. Competitive interaction between these important forage crops is well understood^39^,^49^. We located experimental plots at the University of Guelph Turfgrass Institute in an area homogeneous with respect to micro-topography (43°33′*N*, 80°13′*W*). To minimize spatial heterogeneity, vegetation and the top layer of soil were removed, and the soil was tilled before the experiment began.

### Experimental Design

We established plots with interface length *L* = 1, 2, 4, 8, and 16 *m*, with four replicates of each length. To avert edge effects, we added a 0.5 *m* buffer, where no data were collected, at both ends of every plot. A plot had dimensions of *L* × 3 *m*; all plots were initially split lengthwise by plastic dividers into sections of 1 *m* and the remaining 2 *m*. We planted *T. repens* in the one-meter sections, and *L. perenne* in the two meter sections; we anticipated that clover would advance, given the soil resources and periodic mowing.

In Fall 2007 we planted Dutch white clover seed and a perennial ryegrass mix at respective densities of >1.28 kg/100 *m*^2^, and >7.5 kg/ha. The ryegrass mix consisted of 40% Barclay, 30% Passport, and 30% Goalkeeper varieties. For ease of planting, plots were arranged (with one exception) so that monocultures in one row bordered monocultures of the same species in the next row. Experimental blocks were arranged linearly from the northeast to the southwest. Spatial constraints required two rows within each block, aligned from the northwest to the southeast. One row in each block contained plots of *L* = 1 and 16 *m* side by side, separated by their buffer areas, plus an additional one meter gap to ensure independence of the plots. The other row contained plots of the remaining *L* in the same manner. The order of the rows within the block and the position of plots within a row were randomly selected. Blocking exerted no significant statistical effects on the results.

The area was watered as necessary; the well drained, sandy loam soil prevented excessive moisture accumulation. Ryegrass required fertilization twice before it became fully established (on 7/7/2008 and 9/19/2008; each time we applied 25 kgN/ha). To remove weeds without disturbing the soil, we sprayed herbicide twice (7/7/2008 and 9/19/08). The clover was sprayed with a grass control herbicide (*Poast Ultra*, 1 L/ha), and the grass with a broad-leaf control herbicide (*Par 3*, 55 mL/100 *m*^2^). Throughout the experiment we removed weeds manually, unless removal would disturb the interface.

In spring 2009, monocultures achieved densities sufficient for the experiment. On May 20, plastic barriers between monocultures were removed. Plots were mowed weekly to 5 *cm* above ground through the end of October 2009. There was very little advance of the front in this first season (no movement in most plots) possibly due to the intense mowing regime. In 2010, we mowed only once a month to 8 *cm* above ground, and the clover steadily advanced. We took monthly photos just after mowing.

In June 2010 we began recording the monthly advance of *T. repens* in each plot. We resolved measurements at a scale of 1 *cm*^2^, the size of an individual clover ramet^50^. We marked each 1 *m*^2^ subsection of every plot permanently, to reference growth measurements. Each such subsection was photographed from above after monthly mowing. We re-projected each photo to correct for perspective, and combined photos from the same plot. We recorded the 100 *L* row heights *h*_*y*_(*t*) for *T. repens* in each plot.

We tested the hypothesized power-law relationships against alternative linear and exponential models^51^. We fit power-law models with two different assumptions regarding error distribution. The first assumed normally distributed, additive error; the second assumed log-normally distributed, multiplicative error^52^. Our kinetic-roughening framework predicts the latter. We compared relative support for each model using differences in AIC scores (Δ*AIC*); we considered models with Δ*AIC* < 2 as supported substantially^53^.

After confirming scaling relationships statistically, we compared the exponents to those of the KPZ universality class. We report the slopes ± standard error from all of our power-law scaling results, except for *C*_*t*_(*l*) and *w*(*l*) where we simply provide the estimated exponent. Although we have followed standard kinetic roughening theory protocol, the inherent autocorrelation in these two latter regressions invalidates ordinary least squares confidence intervals. This leaves one estimate with standard error for *β* from interface growth during development, and two estimates of *α* from the interface width and front-runner analyses. We estimated *z* as 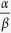 using both *α* estimates and allowing uncertainty to propagate. Finally, we tested all exponents with available standard errors against KPZ values with t-tests.

## Additional Information

**How to cite this article**: Allstadt, A. J. *et al*. Spatial Competition: Roughening of an Experimental Interface. *Sci. Rep.* **6**, 29908; doi: 10.1038/srep29908 (2016).

## Supplementary Material

Supplementary Information

## Figures and Tables

**Table 1:** Definitions of variables.

Symbols	Definitions
*L*_*x*_, *L*_*y*_(=*L*)	Lattice size (*L* = interface length = front length)
*h*_*y*_(*t*)	Rightmost invader in row *y* at time *t*
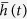	Mean of *h*_*y*_(*t*) (the average is taken across all rows *y*)
*h*_*max*_(*t*)	Rightmost invader at time *t*, front-runner
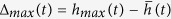	Distance from front-runner to mean of front
〈*w*^2^〉	Mean squared interface width
*ξ*(*t*)	Correlation length along interface
*t*_×_	Crossover time, where *w*^2^ equilibrates
*α*	Roughness exponent
*β*	Growth exponent
*z*	Dynamic exponent
*ρ*_*t*_(*h*)	Mean invader density at height *h*(*t*)

**Table 2:** Expected scaling relationships during development of the interface, expected exponent values for a Kardar-Parisi-Zhang (KPZ) system, and estimated exponents from our field experiment.

Regime	Predicted Scaling	Comment	KPZ Exponent	Field Exponent
*Development*	*ξ*(*t*) ~ *t*^1/*z*^	Correlation length, dynamic exponent	*z* = 3/2	*z* = 1.522 ± 0.708^†^
	*t*_×_ ~ *L*^*z*^	Crossover time, interface length		*z* = 0.810 ± 0.262^‡^
	*w*_*t*_ ~ *t*^*β*^	Interface width, growth exponent	*β* = 1/3	*β* = 0.312 ± 0.073
	*C*_*t*_(*l*) ~ *l*^*α*^	Height-difference correlation, *l* < *ξ*(*t*)	*α* = 1/2	*α* = 0.277
*Stationarity*	*w*(*l*) ~ *l*^*α*^	Local interface width		*α* = 0.311
	*w*(*L*) ~ *L*^*α*^	Interface width		*α* = 0.278 ± 0.181
	〈Δ_*max*_〉_*L*_ ~ *L*^*α*^	Front-runner’s lead		*α* = 0.475 ± 0.191

*z* estimated by *z* = *α*/*β*, with *α* estimates from the front-runner’s lead^†^ and plot interface width^‡^. Of all estimates with standard errors, only *z*^‡^ differs significantly from KPZ.

**Table 3:** Δ*AIC* scores.

Clover GrowthAnalysis	Linear	Exp 1	Exp 2	Pow 1	Pow 2
*w*_*t*_ ~ *t*^*β*^	411.21	407.82	4.65	0	408.72
*C*_*t*_(*l*) ~ *l*^*α*^	2014.94	1716.08	754.1	0	1398.53
*w*(*l*) ~ *l*^*α*^	3629.0	3061.25	1528.87	0	2528.99
*w*(*L*) ~ *L*^*α*^	118.44	117.02	2.56	0	117.18
〈Δ_*max*_〉_*L*_ ~ *L*^*α*^	158.15	155.15	6.48	0	155.76

Models compared are as follows. Linear: *y* = *x* + *ε*; Exp 1: *y* = *log*(*x*) + *ε*; Exp 2: *log*(*y*) = *x* + *ε*; Pow 1: *log*(*y*) = *log*(*a*) + *b* *log*(*x*) + *ε*; Pow 2: *y* = *ax*^*b*^ + *ε. ε* is a random error term with zero expectation and finite variance. Results support power-law models.
