# Supplementary material for: Spatial Competition: Roughening of an Experimental Interface
Source: Sci Rep. 2016 Jul 28;6:29908. doi: 10.1038/srep29908 (PMC4964332; doi:10.1038/srep29908)
Supplement: Supplementary Information [file srep29908-s1.pdf]

Supplement to Spatial Competition: Roughening of an Experimental Interface

Andrew J. Allstadt[1,*], Jonathan A. Newman[2], Jonathan A. Walter[3], G. Korniss[4], and Thomas Caraco[5]

[1]Department of Forest and Wildlife Ecology, University of Wisconsin-Madison, Madison, Wisconsin 53706 USA
[2]College of Biological Science, University of Guelph, Guelph, Ontario N1G 2W1 Canada
[3]Department of Ecology and Evolutionary Biology and Kansas Biological Survey, University of Kansas, Lawrence, KS 66047 USA
[4]Department of Physics, Applied Physics and Astronomy, Rensselaer Polytechnic University, Troy, NY 12308 USA
[5]Department of Biological Sciences, University at Albany, Albany, New York 12222 USA
[*]allstadt@wisc.edu

Supplementary Material

Figure 2 in the main text shows a roughened front generated by simulating preemptive competition. Dispersal was limited to a sites four nearest neighbors; the invader had the greater propagation rate, and the two species had the same mortality rate. The "waviness" of the interface is the result of stochastic roughening. The figure diagrams the width about the mean invasive advance, the front runner's lead, and the correlation length. Note that characterizing the front by each row's height (rightmost position of the invader in each row) ignores "overhangs" where one to a few resident individuals occupy a row to the left of the invader's rightmost position.

Interface profiles show the frequency of invader-occupied sites at each location x, and so suggest how biotic interactions are organized within the interface. From the text, the density profile is:

$$\rho_t(h) = \frac{1}{2}\, erfc\left([h-\overline{h}(t)]/w_t\right) \tag{1}$$

The complementary error function used in the text is:

$$erfc\ (x) = \frac{2}{\sqrt{\pi}}\int_x^{\infty} exp\left[-z^2\right] dz\ . \tag{2}$$

If we neglect open sites (a reasonable approximation for our clover-ryegrass interface) intraspecific interactions will occur at height h in proportion to $[\rho_t(h)]^2$. Their frequency will decline faster than invader frequency within the interface width. Interspecific interactions, if proportional to $\rho_t(h)[1-\rho_t(h)]$, will increase initially, peak at $\overline{h}(t)$, and then decline. Given this approximation, greater roughening increases interspecific mixing at the interface in a quantifiable manner; see Fig. S2. Extending this basic picture, we can ask how interface width affects, and is affected by, ecological detail. For a given invader-resident pair, the increase in equilibrium width, $\langle w^2(L,\infty)\rangle$, with front length L could affect densities of between-species interactions, and so affect the timescale of ecological invasion. Note that if we re-scale each month's height as $[h-\overline{h}(t)]/w_t$, we find a common structure for the density profiles. The final subplot in Fig. S1 (lower right) shows "data collapse" of the last four months' profiles.

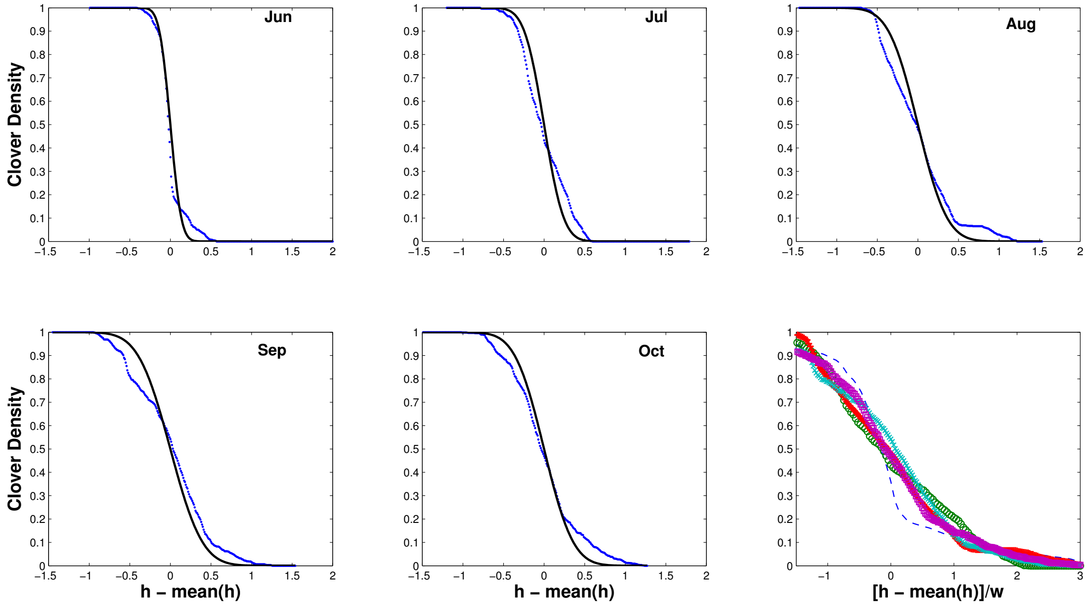

Figure S1. Density profiles of interface width, June through October (as indicated); $L = 16$ m. Each month's empirical profile indicated by (•). Associated complementary error function for each month, parameterized by observed width, approximates data. Interface widths for consecutive month are, respectively, $w = 0.15, 0.27, 0.4, 0.44, 0.4$. Lower right. Data collapse. Dividing height (relative to front's mean position) by width indicates that last four months' clover-density profiles share structural organization. First month (broken line) insufficiently roughened to "fit." Symbols are empty circle (Jul), square (Aug), × (Sep) and closed circle (Oct).

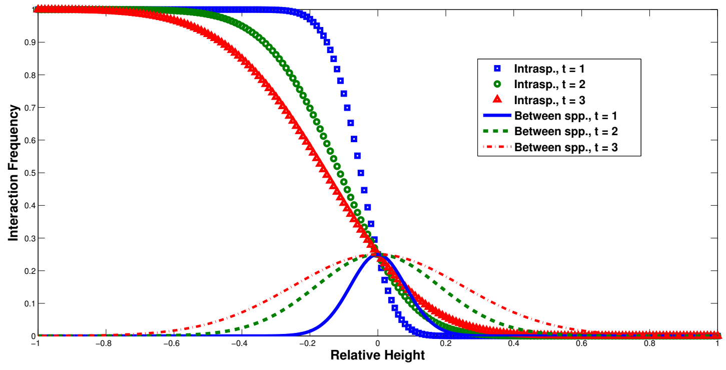

Figure S2. Invader interaction frequencies. $w(t = 1) = 0.15$ (blue), $w(t = 2) = 0.27$ (green), and $w(t = 3) = 0.4$ (red). As time advances, interface width increases. As width increases, (1) decline in intraspecific competition behind mean height exceeds increase in intraspecific competition in front of mean height, and (2) interspecific competition increases symmetrically about mean height.
